# Type 2 Diabetes Mellitus: New Pathogenetic Mechanisms, Treatment and the Most Important Complications

**DOI:** 10.3390/ijms26031094

**Published:** 2025-01-27

**Authors:** Ewelina Młynarska, Witold Czarnik, Natasza Dzieża, Weronika Jędraszak, Gabriela Majchrowicz, Filip Prusinowski, Magdalena Stabrawa, Jacek Rysz, Beata Franczyk

**Affiliations:** 1 Department of Nephrocardiology, Medical University of Lodz, Ul. Zeromskiego 113, 90-549 Lodz, Poland; 2 Department of Nephrology, Hypertension and Family Medicine, Medical University of Lodz, Ul. Zeromskiego 113, 90-549 Lodz, Poland

**Keywords:** diabetes type 2, diabetes complications, pathophysiology of type 2 diabetes, diabetes treatment, cardiovascular disease, stroke, peripheral artery disease, chronic kidney disease, retinopathy, neuropathy, non-alcoholic fatty liver disease in diabetes

## Abstract

Type 2 diabetes mellitus (T2DM), a prevalent chronic disease affecting over 400 million people globally, is driven by genetic and environmental factors. The pathogenesis involves insulin resistance and β-cell dysfunction, mediated by mechanisms such as the dedifferentiation of β-cells, mitochondrial dysfunction, and oxidative stress. Treatment should be based on non-pharmacological therapy. Strategies such as increased physical activity, dietary modifications, cognitive-behavioral therapy are important in maintaining normal glycemia. Advanced therapies, including SGLT2 inhibitors and GLP-1 receptor agonists, complement these treatments and offer solid glycemic control, weight control, and reduced cardiovascular risk. Complications of T2DM, such as diabetic kidney disease, retinopathy, and neuropathy, underscore the need for early diagnosis and comprehensive management to improve patient outcomes and quality of life.

## 1. Introduction

T2DM is a disease of civilization; based on the latest data from the NCD Risk Factor Collaboration (2022), the number of patients was 828 million, of which over 95% had type 2 diabetes [1]. Statistics show that the prevalence of diabetes will continue to rise and by 2050, it will be 10.8% in the United States alone; however, this is likely higher due to varying estimates of the current prevalence of diabetes [1,2,3]. T2DM is a complex, multisystemic metabolic disorder characterized by high blood glucose levels resulting from a progressive defect in insulin secretion or tissue resistance to insulin [4,5]. T2DM is a common and heterogeneous disorder characterized by varying degrees of beta-cell dysfunction and insulin resistance. There is a strong association between obesity and T2DM, involving pathways regulated by the central nervous system. These pathways control food intake and energy expenditure, integrating information from peripheral organs and the environment [6]. It should also be mentioned that T2DM is not only a domain of older people; in recent years, in younger people (<40 years of age), a two- or three-fold increase in the incidence of T2DM has been noted [7]. In the population of young people, in order to correctly diagnose and recognize T2DM, it is necessary to exclude other types of diabetes, which may give a similar medical sign. In the differentiation of T2DM in young people, we consider type 1 diabetes mellitus (T1DM), latent autoimmune diabetes of adults (LADA), and maturity onset diabetes of the young (MODY). Establishing the correct diagnosis is very important in terms of prognosis, possible complications, and initiating appropriate treatment [7]. The criteria for diagnosing T2DM (Table 1) in non-pregnant adults according to the American Diabetes Association (ADA) guidelines include hemoglobin A1C (HbA1C) ≥ 6.5%, fasting plasma glucose ≥ 126 mg/dL or plasma glucose after a 2 h oral glucose tolerance test (OGTT) at a dose of 75 g of ≥200 mg/dL, and plasma glucose in a random test of ≥200 mg/dL with simultaneous symptoms of hyperglycemia or hyperglycemic crisis; in the case of an equivalent result, the tests should be repeated [8]. In this article, we would like to present in a clear and understandable way new mechanisms that play a role in the pathogenesis of T2DM, as well as available treatment methods based on the latest guidelines with a focus on possible complications.

## 2. Pathophysiology of T2DM

T2DM has a multifactorial etiology, a combination of genetic and environmental factors [9]. The main pathomechanisms in which T2DM is developed are the defect of insulin production and insulin resistance (IR) in peripheral tissues [9]. The dysfunction of pancreatic β-cells causes a reduction in insulin secretion that results in the inability to maintain physiological glucose levels, while IR promotes the production of glucose in the liver and decreases glucose uptake in muscle, liver, and adipose tissue, thus creating a flawed feedback loop between insulin action and secretion, leading to hyperglycemia [10,11].

### 2.1. β-Cell Dysfunction

**Figure 1:**
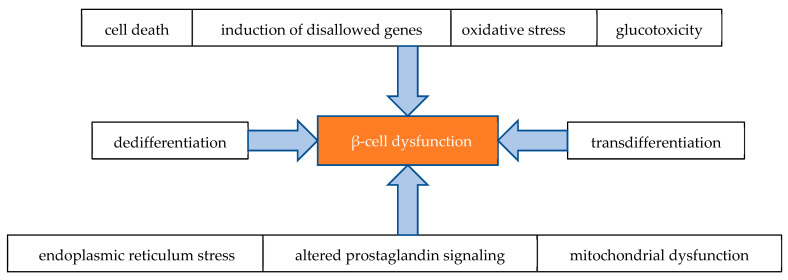
Main mechanisms contributing to β-cell dysfunction.

Traditionally, the dysfunction of β-cells has been attributed to the loss of β-cell mass due to β-cell exhaustion in a state of prolonged elevations in glucose metabolism and insulin secretion, as well as β-cells apoptosis caused by glucotoxicity and lipotoxicity [12,13,14,15]. However, it is suggested that the impaired function of β-cells may be a result of more complex mechanisms and interactions, which are depicted in Figure 1 [14].

One of the proposed mechanisms is the dedifferentiation of β-cells, a process defined as the loss of β-cell-defining transcription factors [16]. Such loss of identity of a β-cell can occur as a result of glucotoxicity [17]. A study by Guo [18] et al. has shown that β-cells from mice T2DM models and humans with T2DM lost transcription factors associated with mature β-cells, such as Pdx1 and MafA. Moreover, the inactivation of MafA has been associated with impaired β-cell glucose-stimulated insulin secretion [19].

Another mechanism is the transdifferentiation of β-cells, which is a process of converting one terminally differentiated cell type into another [20]. A study by [21] on the T2DM mice model has found polyhormonal cells in the pancreatic tissue, which might suggest transdifferentiation. Another study by [22] on mice has found that the deletion of transcription factor Nkx2.2 in β-cells resulted in the induced expression of other non-β cell endocrine features and the creation of reprogrammed and bihormonal cells, while simultaneously causing the onset of a diabetic phenotype in these mice. A study by Gao et al. [23] has shown that the loss of Pdx1 transcription factor in β-cells resulted in β-cells acquiring α-cell physiological features. Similarly, a different study by [20] has found that β-cells can transdifferentiate into glucagon-secreting α-cells. A study by Cinti et al. [24] has examined pancreatic islets from diabetic and nondiabetic organ donors and found that β-cells in humans with T2DM become dedifferentiated and convert to α- and δ-“like” cells. A study by Spijker et al. [25] has provided more evidence that the loss of β-cells identity and their conversion into α-cells can occur in vivo, and this is associated with the presence of islet amyloidosis and diabetes incidence. While more studies on humans are needed, the identification of mechanisms that trigger the loss of human β-cell identity may propose new strategies of preventing and delaying the progression of T2DM [25].

β-cells’ function can be impaired through the induction of disallowed genes as well [16]. Disallowed genes are genes that are upregulated in the state of metabolic stress, such as T2DM, while the proper markers of β-cells are downregulated [16]. These include genes that are repressed in pancreatic cells but expressed widely in cells of different tissues [26]. There are several genes proposed as the disallowed genes, including a gene encoding repressor element 1 silencing transcription factor (REST), whose repression is necessary for a physiological secretion of insulin [16]. The overexpression of REST has been associated with lower functional β-cell mass and diabetes [27]. The overly expressed REST leads to the activation of expression of dual-specificity tyrosine-regulated kinase 1A (DYRK1A), a kinase involved in the repression of β-cell proliferation, thus resulting in impaired β-cell compensation in the state of T2DM [27,28]. More studies are needed to understand the expression of the disallowed genes in β-cells [16]. It is speculated that mechanisms such as histone modifications, DNA methylation, and microRNAs are involved in this process [16].

Chronic hyperglycemia can result in glucotoxicity which promotes the development and progression of T2DM [29,30]. Elevated levels of NADH and reactive oxygen species (ROS), which are present in chronic hyperglycemia, have been associated with the dysfunction of β-cell [31]. However, some of the effects of glucotoxicity on cells may be reversible in the mechanism of β-cell reset [32]. For instance, studies on humans with T2DM who underwent bariatric surgery have shown that it helps restore a normal glucose-stimulated insulin secretion and normalize blood sugar [32,33,34].

Mitochondrial dysfunction is another factor that can lead to β-cell dysfunction, as seen in T2DM, and it has been observed that mitochondria in humans with T2DM are smaller, fragmented, and swollen [31,35,36,37]. Mitochondria are a source of ROS, which β-cells are very sensitive to due to their low levels of antioxidant enzymes and high oxygen consumption [16,38]. ROS in small amounts exert a beneficial effect of stimulating insulin expression and are necessary for glucose-induced insulin secretion [39]. However, in larger amounts, ROS can lead to β-cell dysfunction and death [37]. A study by Fu et al. [40] on a β-cell line has found that chronic exposure to high glucose and palmitate, which were used to mimic glucolipotoxicity, was associated with greatly reduced insulin secretion and elevated levels of ROS. It is speculated to be a cause of defected expression and activity of MafA in the state of oxidative stress [18].

Thioredoxin-interacting protein (TXNIP), which is a factor associated with oxidative stress and glucotoxicity, has been found in elevated levels in prediabetic and T2DM patients [41,42]. TXNIP has been shown to promote β-cell apoptosis, while its deletion has been found to promote insulin production and glucagon-like peptide 1 signaling [42]. Therefore, data suggest that TXNIP may be a new therapeutic target for T2DM [42].

The state of hyperglycemia and increased insulin production can lead to endoplasmic reticulum (ER) stress, which can result in unfolded protein response (UPR) in β-cells [43,44]. Unfolded protein response is a compensatory reaction that inhibits protein production to allow for the refolding or degradation of improperly folded proteins [45,46]. Yet, prolonged and uncontrolled UPR can lead to the upregulation of CHOP, which is a protein involved in apoptosis, and therefore, increased cell death [16]. A study by Arunagiri et al. [47] has shown that the accumulation of misfolded proinsulin leads to exacerbated ER stress, UPR, decreased synthesis of insulin, hyperglycemia, and diabetes. Moreover, the increased accumulation of islet amyloid polypeptide in β-cells is also suggested as a factor leading to ER stress and the development of T2DM [48].

Systemic inflammation and hyperglycemia, which are present in T2DM, can lead to alterations in prostaglandin signaling [49]. One of the upregulated signaling molecules in T2DM is prostaglandin E2 (PGE2), which binds to a few different receptors, including EP2 [49,50]. EP2 expression is elevated in T2DM, which suggests that its activity contributes to defects in the compensatory mechanism of β-cells [49]. Moreover, it has been found that the blockade of EP2 leads to enhanced β-cell proliferation and survival, proposing it as a new treatment target for T2DM [49,50].

### 2.2. Insulin Resistance (IR)

IR means a decline in a target cell’s metabolic response to insulin or, at a systemic level, a decreased blood glucose-lowering effect of insulin [51]. IR can be a result of decreased insulin secretion, insulin antagonists in the plasma, and diminished insulin response in target tissues [52]. The action of insulin is regulated by different hormones, including growth factors and insulin-like growth factor 1 (IGF-1) in the fed state and glucagon, glucocorticoids, and catecholamines in the state of fasting [53,54]. Therefore, the immoderate production of these hormones may promote IR [53,54]. The balance between insulin and glucagon is especially important since it determines the relative degree of phosphorylation of downstream enzymes in the regulatory signaling pathways [53,54].

**Figure 2:**
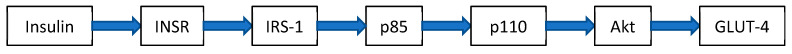
Insulin signal transduction in a normal glucose-tolerant state.

IR of skeletal muscles is regarded as one of the most essential extra-pancreatic factors in the development of T2DM [55]. In the physiological state, insulin promotes the production of glycogen in skeletal muscle via glucose uptake from plasma [56]. One of the most important factors in this process is glucose transporter type 4 (GLUT4), which translocates from intracellular compartments upon insulin binding to the insulin receptor (INSR) in muscle cells, which allows for glucose uptake as depicted in Figure 2 [57]. Therefore, any mutations that reduce the expression of INSR or GLUT4 and all defects in upstream or downstream signaling pathways can result in reduced glucose intake into the muscle and thus a hyperglycaemic state [51]. Moreover, mutations of INSR tyrosine kinase, which allows for insulin-mediated signaling, and any key proteins of the downstream signaling pathway such as insulin receptor substrate 1 (IRS-1) and insulin receptor substrate 2 (IRS-2) or phosphoinositide 3-kinase (PI3K) could also lead to an impaired insulin effect on the muscle tissue [51,58]. Environmental factors also play a role in glucose intake; in obesity, increased immune cell infiltration and the secretion of proinflammatory molecules can result in skeletal muscle inflammation, thus leading to myocyte inflammation, impaired myocyte metabolism, and the promotion of IR via paracrine effects [59]. On the contrary, physical activity is known to increase the blood flow into the muscle, which leads to enhanced glucose uptake and thus reduced IR [60].

An impaired response to insulin by adipose tissue can result in compromised suppression of lipolysis, impaired glucose uptake, and enhanced free fatty acid (FFA) release into the plasma even in the presence of high insulin levels [61]. Moreover, the accumulation of FFA in the liver can lead to compromised insulin signaling and, thus, the promotion of hepatic gluconeogenesis and impaired glucose-stimulated insulin response, which can induce the development of T2DM [51]. Studies have also shown that the defective activation of protein kinase B (AKT) promotes the lipolytic enzymes that further worsen hyperglycemia [51]. Furthermore, increased adipose tissue mass, such as in obesity, has been associated with pathologic vascularisation, hypoxia, fibrosis, and inflammation [62]. Obesity and high-fat diets have been shown to be able to promote the activation of saturated FFA-stimulated adenine nucleotide translocase 2 (ANT2), an inner mitochondrial protein that promotes adipocyte hypoxia and the activation of the transcription factor hypoxia-inducible factor-1α (HIF-1α), thus causing adipose tissue dysfunction and inflammation [62,63]. Hypertrophied adipocytes are also responsible for elevated levels of proinflammatory cytokines, which result in a chronic state of low-grade systemic inflammation, also referred to as metabolic inflammation [63]. The aforementioned state of metabolic inflammation is regarded as an essential factor in the pathogenesis of IR and T2DM [64].

In the liver, insulin partakes in regulating glucose production and utilization, and it affects lipid metabolism via different downstream pathways that regulate multiple metabolic processes, such as glycogen synthesis, gluconeogenesis, glycolysis, and lipid synthesis [65]. The regulation of hepatic glucose output is achieved via the combined action of glucagon and insulin; glucagon promotes the synthesis of glucose, while insulin inhibits it if serum glucose is elevated [66]. Moreover, insulin promotes the activation of transcription factor forkhead box protein O1 (FOXO1), which leads to the inhibition of key enzymes for gluconeogenesis [67]. Thus, insulin promotes the storage of glucose as glycogen and inhibits glucose synthesis and glucose output [67]. However, in the state of IR, the levels of circulating insulin are not sufficient to exert an appropriate insulin response in hepatic cells [68]. In the liver, IR diminishes the production of glycogen, fails to suppress glucose production, and promotes lipogenesis and the synthesis of proinflammatory proteins [67]. The excessive synthesis of such proinflammatory cytokines in the state of oxidative stress can result in a systemic inflammatory state that is responsible for impaired insulin response induced by the liver [67].

### 2.3. Role of Gut Microbiota

Evidence suggests that gut microbiota partake in the pathophysiology of multiple chronic diseases, T2DM included [69]. A study by Gurung et al. [69], after summarizing 42 human studies on microbial associations with T2DM, has found that the genera of *Ruminococcus*, *Fusobacterium*, and *Blautia* were positively associated with T2DM, while the genera of *Bifidobacterium*, *Bacteroides*, *Faecalibacterium*, *Akkermansia*, and *Roseburia* were negatively associated with T2DM. Gut microbiota can contribute to the development of T2DM through multiple molecular mechanisms [70]. For instance, studies have found that microbes such as *Fusobacterium nucleatum* and *Ruminococcus gnavus*, which are associated with T2DM, partake in increasing the synthesis of inflammatory cytokines, which play a role in the development of diabetes, as well as in other diseases like colorectal cancer and inflammatory bowel disease [71,72]. However, gut microbiota can influence T2DM positively as well; for example, it has been shown that *Lactobacillus gasseri* BNR17 increases the expression of GLUT-4 in the muscle, thus exerting a potential anti-diabetes effect [73]. Moreover, gut microbiota can also influence the development of T2DM via microbial metabolites [74]. These metabolites include short-chain fatty acids (SCFAs) such as acetate, propionate, and butyrate [74]. SCFAs have been observed to improve glucose metabolism via the activation of intestinal gluconeogenesis and exert insulin-sensitizing effects as well [75,76]. SCFAs have been found to improve systemic inflammation via the suppression of proinflammatory cytokines and the infiltration of immune cells into adipose tissue, as well as the promotion of anti-inflammatory cytokines [77,78,79]. Branched SCFAs (BSCFAs), such as isobutyric, isovaleric, and methylbutyric acids, are microbial metabolites produced via the fermentation of the branched-chain amino acids [80]. A study by Aslamy et al. [81] has found that a high level of BSCFAs in the blood is associated with a lower prevalence of dyglicemia and improved glucose homeostasis. Furthermore, microbial metabolites such as bile acids and indole derivatives seem to be positively correlated with improved glucose metabolism and a lower risk of T2DM [74]. However, metabolites such as trimethylamine, branched-chain amino acids (BCAAs), and imidazole propionate are suggested to play a role in the pathogenesis of T2DM [74]. While evidence supports the claim that gut microbiota is an important factor in glucose metabolism and the development of diabetes, more studies are needed to properly utilize that knowledge [69].

### 2.4. Role of Fat Mass

Excessive caloric consumption and a positive energy balance lead to the increased accumulation of lipids, obesity, and related comorbidities [82]. A dysfunction of long-term fat storage in the white adipose tissue, due to the inability of subcutaneous adipose tissue to expand properly through hyperplasia, can lead to increased cardiometabolic risk and obesity-related diseases such as T2DM [83]. Evidence suggests that the distribution of fat mass is an important factor in overall metabolic health, which studies defined as an increased gluteofemoral and leg fat mass, together with high insulin sensitivity and high insulin secretion [83,84]. On the other hand, an increased visceral fat mass, increased subcutaneous abdominal fat mass, and high liver fat content can be connected to a higher cardiometabolic risk [83,84]. Increased visceral fat mass has been linked with dysregulated adipokine secretion, inflammation, increased levels of fatty acids in the blood, and ectopic lipid deposition in organs such as the pancreas, liver, and muscles, thus increasing the risk of T2DM [83,85]. Subcutaneous abdominal adipose tissue is divided by the Scarpa’s fascia into superficial subcutaneous adipose tissue, which has a favorable metabolic profile, and deep subcutaneous adipose tissue, which is a strong independent risk factor of insulin resistance because of its high expression of proinflammatory, lipogenic, lipolytic genes and its high content of saturated fatty acids [83,86]. Evidence suggests that deep subcutaneous adipose tissue expands more significantly with an increase in total body mass, making it the prevalent subcutaneous abdominal adipose tissue in obese patients [86].

Moreover, the distribution of fat mass is also crucially important in patients with normal weight, as findings suggest that a lipodystrophy-like phenotype exists in the general population [84]. Studies have found that normal-weight patients who are metabolically unhealthy have higher visceral fat mass, liver fat content, and lower subcutaneous leg fat mass [84]. Normal-weight patients with a lipodystrophy-like phenotype are also strongly characterized by insulin resistance and impaired secretion of insulin [84].

Obesity is often measured using body mass index (BMI), which is an approximation of fat mass [87]. Evidence suggests that height can significantly interact with the correlation between BMI and total fat mass, as the positive relationship between BMI and total body fat mass becomes stronger with increasing height [87]. A study by Wittenbecher et al. [88] has found that higher adult height is linked to a lower risk of T2DM. However, a study by Stefan et al. [87] has shown a highly significant interaction between height and BMI on the prevalence of T2DM. These findings suggest that BMI better reflects fat mass and cardiometabolic risk in people of higher height compared with shorter individuals [87]. Moreover, since people nowadays are taller on average, they face a larger BMI-associated health burden for a similar BMI than people in the past [87]. Therefore, it is important to account for height changes over the past to improve the estimation of the burden of cardiometabolic diseases associated with obesity [87].

## 3. Pharmacological Methods of Treatment

### 3.1. SGLT2 Inhibitors

Relatively new medications used in the treatment of T2DM include SGLT2 inhibitors (SGLT2is), also known as gliflozins. Sodium–glucose cotransporter 2 (SGLT2), found in the proximal tubule of the kidney, plays a key role in glucose reabsorption by moving glucose from the lumen of the renal tubule into the epithelial cells lining the tubule [89]. An SGLT2i works by blocking the activity of this protein, which results in lower glucose levels in the bloodstream [89]. Most of SGLT2i compounds are predominantly selective for SGLT2, found in the renal proximal tubules, with a selectivity that is 200–2500 times greater compared to SGLT1, which is present in both the kidneys and the gastrointestinal tract [90]. Clinical studies of SGLT2 inhibitors have consistently demonstrated their effectiveness in lowering blood glucose levels, with reductions in HbA1c ranging from 0.5 to 0.9% (5–9 mmol/mol) after 12 months of therapy. Additionally, the glucoretic effect contributed to a clinically meaningful decrease in systolic blood pressure (SBP) by approximately 2.5–5.0 mm Hg and an average weight loss of about 2 kg [91]. Meta-analyses of clinical studies involving patients using SGLT2 inhibitors have demonstrated notable reductions in body weight. This effect is primarily linked to caloric loss and a metabolic shift from glucose utilization to ketone and fatty acid metabolism. This transition promotes increased fat burning, thereby contributing to weight loss [91,92,93]. Treatment with these medications can be initiated when the estimated glomerular filtration rate (eGFR) exceeds 60 mL/min/1.73 m^2^ and should be re-evaluated if it decreases to 45 mL/min/1.73 m^2^, as the glucose-lowering efficacy of SGLT2i is mainly dependent on renal function [76]. SGLT2is are advised as a component of holistic treatment plans, as they not only help lower blood sugar levels but have also been shown in numerous studies to decrease the risk of chronic kidney disease (CKD) progression and cardiovascular disease (CVD) complications [94,95,96]. Genital infections are a notable side effect of SGLT2i therapy, primarily resulting from glucosuria, which provides an environment conducive to pathogen growth. The main etiological agents of these infections, associated with flozins, are fungi. These infections are more commonly observed in women (10%) compared to men, where the incidence ranges from 2% to 3% [97,98]. Other rare side effects associated with flozins include diabetic ketoacidosis, particularly among patients using insulin or undergoing surgical procedures [91]. Additionally, an increased incidence of lower limb amputations has been reported in patients treated with canagliflozin [95].

### 3.2. GLP-1 Receptor Agonists

Glucagon-like peptide (GLP-1) receptor agonists are newly approved medications for the treatment of diabetes and obesity. GLP-1 is an intestinal peptide secreted by epithelial L-cells in response to nutrient intake, particularly glucose and lipids. GLP-1 exerts physiological effects on various organs. As an incretin, it enhances glucose-dependent insulin secreted by pancreatic β-cells, promotes β-cell neogenesis, inhibits β-cell apoptosis, and suppresses glucagon secretion from α-cells (as observed in rodent studies). Additionally, GLP-1 influences other tissues and organs, including the stomach by delaying gastric emptying, the heart by exerting cardioprotective effects, and adipose tissue and skeletal muscle by improving glucose uptake, and it also acts centrally on neurons in the hypothalamus, inducing a feeling of satiety [99,100,101]. Therapy with GLP-1 receptor agonists suppresses appetite, resulting in weight loss. This, in turn, has a broad impact on improving patients’ quality of life and reducing the risk of cardiovascular and renal complications [102]. Various GLP-1 receptor agonists have been authorized for managing T2DM, including exenatide, liraglutide, lixisenatide, dulaglutide, and semaglutide. These medications are primarily administered via subcutaneous injection, although an oral formulation of semaglutide is also available [102]. Scientific studies have demonstrated that GLP-1 analogs improve glycemic control in patients with T2DM and additionally contribute to a reduction in SBP. Long-acting agents within this drug class are associated with more effective glucose lowering and exhibit fewer gastrointestinal side effects compared to their short-acting counterparts [103,104,105,106]. Given that GLP-1 analogs are a relatively new and insufficiently studied class of drugs, their adverse effects are not yet fully understood. However, among the side effects identified so far, nausea and vomiting are the most common. Furthermore, nasopharyngitis and headaches associated with injections may occasionally occur [107].

### 3.3. DPP-1 Inhibitors

After the discovery of GLP-1, targeting DPP-4 inhibition became a key focus in research. Blocking DPP-4 significantly impacts incretin hormone activity by raising the levels of endogenous active peptides in the bloodstream [108]. The primary effects associated with DPP-4 inhibition are attributed to elevated GLP-1 levels. As a result, DPP-4 emerged as an important target for managing T2DM [109]. The DPP-4 enzyme, found extensively in endothelial cells, the immune system, and various other tissues, plays a crucial role in glucose metabolism by deactivating incretin hormones such as glucagon-like peptide-1 (GLP-1) and glucose-dependent insulinotropic polypeptide (GIP). Blocking DPP-4 prolongs the half-life of GLP-1, enhancing insulin release and reducing glucagon secretion in a glucose-dependent way. Beyond their role in regulating glucose, GLP-1 and GIP also have cardiovascular benefits, including enhancing endothelial function, reducing oxidative stress, and providing anti-inflammatory effects, which may offer advantages in the treatment of heart failure [110,111]. To date, five DPP-4 inhibitors, known as gliptins, have been approved for clinical use: sitagliptin, vildagliptin, linagliptin, saxagliptin, and alogliptin. These medications share a similar mechanism of action but differ in their pharmacokinetic properties. Sitagliptin and alogliptin are primarily eliminated through renal excretion, whereas hepatic metabolism is the main pathway for saxagliptin elimination. Linagliptin, on the other hand, is predominantly excreted via the biliary route [112]. The most common adverse effects associated with DPP-4 inhibitor therapy include nasopharyngitis, skin rash, and mild gastrointestinal disturbances [113]. Additionally, this class of drugs has no significant impact on body weight. DPP-4 inhibitors increase GLP-1 levels by two- to three-fold, compared to a ten-fold increase observed with GLP-1 receptor agonists. Despite their numerous benefits, DPP-4 inhibitors are less effective in reducing HbA1c levels compared to GLP-1 receptor agonists [114].

### 3.4. Tirzepatide

Tirzepatide is a novel medication that leverages the dual agonism of glucose-dependent insulinotropic polypeptide (GIP) and GLP-1 receptors, resulting in improved blood glucose control and significant weight reduction [115,116]. Its affinity for the GIP receptor is equivalent to that of endogenous GIP, while its affinity for the GLP-1 receptor is five times lower than that of endogenous GLP-1 [117,118]. Additionally, tirzepatide exerts beneficial effects on blood pressure (BP), LDL cholesterol, and triglyceride levels, suggesting a potential role in reducing the risk of complications associated with T2DM [119,120,121].

In 2021, the SURPASS-1 trial—a randomized, double-blinded clinical study—was conducted to evaluate the efficacy of tirzepatide administered via weekly subcutaneous injections compared to placebo in patients with T2DM inadequately controlled by diet and exercise alone. The study demonstrated that tirzepatide, at all tested doses, was significantly more effective than placebo in reducing body weight, fasting serum glucose, and HbA1c levels. A summary of the SURPASS-1 study outcomes is presented in Table 2 [122]. 

In the SURPASS-1 trial, tirzepatide demonstrated remarkable efficacy in glycemic control compared to placebo, and this led to significant weight loss without an increased risk of hypoglycemia. Its safety profile aligned with that observed for GLP-1 receptor agonists [122].

In 2021, a study comparing the efficacy of once-weekly tirzepatide and semaglutide in patients with type 2 diabetes was conducted. A total of 1879 participants were randomized into four study groups, receiving either tirzepatide at doses of 5 mg, 10 mg, or 15 mg or semaglutide at a dose of 1 mg. The study demonstrated that the groups treated with tirzepatide achieved greater reductions in HbA1c levels and body weight compared to the semaglutide group. Additionally, the tirzepatide groups showed improvements in blood pressure reduction and lipid profile. Adverse events observed in both treatment groups were similar, primarily involving mild to moderate gastrointestinal symptoms [123].

## 4. Non-Pharmacological Methods of Treatment

Non-pharmacological approaches should be an important part of the treatment of T2DM. Pharmacological approaches may be included when lifestyle modification alone is not sufficient to achieve positive results [124]. Recent research supports a holistic, integrative approach to managing T2DM, combining pharmacological treatments with lifestyle changes and psychosocial interventions. In the following paragraph, non-pharmacological methods of treating and supporting the healing process of T2DM will be discussed (Table 3).

### 4.1. Exercise

Physical activity may be important in the treatment of T2DM. Importantly, recent research shows that in addition to the activity itself, its type is also important. Regular resistance exercise has been shown to improve glycemic control, insulin sensitivity, and muscle function in individuals with T2DM. Combining aerobic and resistance exercises appears to be more effective than single-mode training in managing blood glucose levels and enhancing overall metabolic health [125]. Interestingly, studies comparing a group of T2DM patients taking metformin and a group not taking metformin during the 12-week inter-day concurrent training program showed similar effectiveness in improving metabolic markers in patients with IR as the metformin treatment alone. Both exercise groups demonstrated a significant reduction in insulin sensitivity and an increase in maximal fat oxidation [126].

### 4.2. Dietary Interventions

Low-calorie high protein diets improved glucose metabolism and other cardiometabolic outcomes, regardless of protein source (either animal or plant sources), in outpatients with prediabetes or T2DM [127]. The meta-analysis showed that the Mediterranean diet is an effective form of dietary intervention in improving glycemic control, and the low-carbohydrate diet obtained the highest result in anthropometric measurements in people with T2DM and comorbid overweight/obesity [128].

### 4.3. Bariatric Surgery

T2DM often co-occurs with obesity. Bariatric surgery is believed to be effective in treating both T2DM and obesity [129]. It is evident that surgeries like sleeve gastrectomy, one-anastomosis gastric bypass, and Roux-en-Y gastric bypass have the potential to induce remission of T2DM. Factors such as age, baseline BMI, HbA1c, the use of antidiabetic medication, and the duration of diabetes play a major role in T2DM remission alongside the choice of bariatric surgery [130]. Bariatric surgery is less likely to result in remission in patients with a history of insulin therapy and longer durations of T2DM prior to the surgery [131].

### 4.4. Behavioral and Psychological Interventions

Psychological factors can significantly impact the management of T2DM. Recent studies have emphasized the importance of integrating psychological interventions with standard diabetes care. Cognitive-behavioral therapy (CBT) has proven to be an effective treatment for patients with diabetes. The results of a meta-analysis indicate a significant reduction in HbA1c, fasting blood glucose, and diastolic blood pressure (DBP) in patients with diabetes on CBT [132]. Results from a meta-analysis indicated that behavioral strategies had a better effect on glycemic control, and cognitive strategies had a better effect on depressive symptoms. Among the techniques used, the advantages of interventions that emphasized homework assignments, stress management, and interpersonal strategies were particularly important [133]. Another form of psychological support for patients with T2DM is mindfulness-based stress reduction (MBSR). Although the meta-analysis found no effect of MBSR on HbA1C post-intervention or at follow-up, the results suggest that MBSR appears to be an effective treatment for improving mental health and mindfulness in individuals with T2DM [134].

### 4.5. Hyperbaric Oxygen Therapy

Most studies have demonstrated a decrease in blood glucose levels after hyperbaric oxygen therapy (HBOT) in patients with T2DM. Additionally, some research has indicated a significant reduction in HbA1c following HBOT. The mechanism underlying the decrease in blood glucose levels from HBOT seems to be primarily linked to improved insulin sensitivity rather than an increase in insulin secretion [135]. HBOT is also described in the context of treating limb ulcers in patients with T2DM. The results of several studies suggest low or moderate recommendation values, but further research on this topic is needed [136,137].

### 4.6. Probiotics

The consumption of probiotics and synbiotics has positive effects on the glycemic profile of people with prediabetes and T2DM [138]. Probiotics treatment may reduce glycated hemoglobin A1c (HbA1c), fasting blood glucose (FBG), and insulin resistance level (HOMA-IR) in T2DM patients [139]. Additionally, the intake of probiotics or synbiotics may serve as an effective intervention to enhance cardiometabolic health by reducing inflammation and oxidative stress in individuals with prediabetes and T2DM [140].

**Table 3:** Non-pharmacological methods of treatment for T2DM [124,126,129,131,135,139]. T2DM indicates type 2 diabetes mellitus; HbA1c, hemoglobin A1C.

Non-Pharmacological Methods of Treatment T2DM	
Type of Method:	Positive Effects of Therapy:
Exercise	Improvements in glycemic control, insulin sensitivity, and muscle function.
Dietary Interventions	Improvements in glucose metabolism and cardiometabolic outcomes.
Bariatric Surgery	Possibility of remission and weight loss in obese patients with T2DM.
Behavioral and Psychological Interventions	Reductions in HbA1c, fasting blood glucose, and improvements in mental health.
Hyperbaric Oxygen Therapy	Possibility of reductions in blood glucose levels and HbA1c.
Probiotics	Improvements in glycemic profile and cardiometabolic health.

## 5. Complications of T2DM

### 5.1. DKD

Diabetic kidney disease (DKD) is a microvascular complication of DM [141], developing in 40% of people with T2DM [142]. It is the most common form of CKD [143] and the cause of 50% of end-stage renal disease (ESRD) worldwide [144]. It is the strongest risk factor for mortality in DM patients [145]. The risk of death from CVD in people with DKD and T2DM is 13% higher on a 10-year basis compared to T2DM without DKD. [146] In the course of DKD, renal function is impaired, or albuminuria occurs [147]. Tests for DKD in patients with T2DM should be performed at the time of diagnosis of DM [148]. Primary prevention includes the appropriate control of glycemia, hypertension, the treatment of dyslipidemia, and lifestyle modifications [149]. Drugs used in DKD include SGLt2i, GLP-1, dipeptidyl peptidase 4 (DPP4) inhibitors, statins, and angiotensin-converting enzyme inhibitors (ACEIs) [150].

### 5.2. Diabetic Retinopathy

The most common cause of ocular vascular disease is diabetic retinopathy (DR) [151]. In 2020, DR occurred in 103 million people, and this number may reach 130 million in 2030 and even 161 million in 2045 [152]. As many as 60% of people with T2DM will develop a complication in the form of DR after 20 years of disease duration [153], and it is the duration of the disease that is the most important factor in its development [154]. In people suffering from T2DM, the complication of DR occurs three times less frequently compared to T1DM [122]. It occurs in 30–40% of patients with DM [124]. DR is more common in women, but its course is worse in men [155]. It is the fifth cause of vision loss in the world [156]. Symptoms that may accompany this complication include blurred vision, distorted vision, and the partial or complete loss of vision [157]. Techniques such as pars plana vitrectomy (PPV), panretinal laser photocoagulation (PRP), and intravitreal anti-vascular endothelial growth factor (anti-VEGF) injections have been used in the treatment of DR [158].

### 5.3. Neuropathy

The most common complication of diabetes is neuropathy [159]. Neuropathy occurs in almost 45% of T2DM patients [160]. In the course of DM, we can distinguish the following forms of neuropathy: distal symmetric polyneuropathy, autonomic neuropathy, radiculo-plexopathy, and mononeuropathy [161]. Distal symmetric polyneuropathy is the most common form of neuropathy in DM [162]. Symptoms occurring in the course of neuropathy include pain, tingling, paresthesia, numbness, and increased sensitivity to stimuli [163]. Appropriate glycemic control helps prevent or slow down the development of the disease [164]. Testing for this complication should take place at the time of diagnosis of T2DM [165]. In symptomatic treatment, painkillers such as gabapentin, pregabalin, tricyclic antidepressants (TCAs), venlafaxine, and duloxetine are used [166].

### 5.4. Metabolic Dysfunction-Associated Steatotic Liver Disease (MASLD)

In the pathogenesis of MASLD, the excessive consumption of glucose, fructose, and saturated fatty acids is important, which leads to insulin resistance, inflammation, and oxidative stress in the liver, which in turn promotes the development of liver cirrhosis. Additionally, changes in the intestinal microbiome and disorders in the release of adipokines and cytokines from inflamed adipose tissue enhance this pathogenic process [167]. Additionally, changes in the intestinal microbiome and disorders in the release of adipokines and cytokines from inflamed adipose tissue enhance this pathogenic process (the one they gave in the review). In the pathophysiology of MASLD, in addition to global metabolic mechanisms, intrahepatic pathways play a key role. Various genetic variants have been identified, such as PNPLA3, TM6SF2, MBOAT7, GCKR, and HSD17B13, which regulate triglyceride mobilization, VLDL secretion, and processes related to lipogenesis and lipid remodeling [168,169]. Risk factors include obesity, type 2 diabetes, hypertriglyceridemia, and metabolic syndrome [170]. The treatment of this disease includes lifestyle modifications, diet, and antidiabetic drugs [171].

### 5.5. Coronary Artery Disease

People with diabetes predominantly experience mortality due to ischemic heart disease about two to four times more frequently compared to people free of diabetes. Additional risk elements, universally applicable across populations, include hypertension, hypercholesterolemia, the presence of microvascular complications, advanced age, sex, smoking status, glycemic control, and elevated body mass index (BMI) [172]. The appearance of one or more of these risk factors, however, may result in a worse quality of life for people with diabetes than for people without it [173].

Ischemic heart disease is characterized by an imbalance between myocardial oxygen supply and demand, leading to compromised blood flow and subsequent myocardial injury [174]. This condition is strongly linked to coronary artery disease (CAD), whose initial clinical manifestation may present as an acute myocardial infarction resulting from the disruption of atherosclerotic plaques, leading to the obstruction of the coronary vessels [175]. The hallmark symptoms include angina and chest pain, typically described as a pressure or discomfort located retrosternally, which may radiate to the jaw, shoulder, or arm. Other signs of CAD include dyspnea, diaphoresis, fatigue, nausea, and lightheadedness [176].

### 5.6. Stroke

Stroke, as a major component of CVD, poses a significant healthcare challenge not only for developing countries but also for developed ones, with far-reaching consequences for patients’ health and lives. Additionally, it incurs substantial cost for society, estimated at $273–818 billion in the United States alone [177]. Patients with diabetes, according to data from the Greater Cincinnati/Northern Kentucky stroke study, are more likely to suffer from ischemic stroke incidents in every age group than patients without this disease, especially before the age of 65 in Whites and 55 in African Americans [178]. It has been discovered that prediabetes may also be a cause of higher frequency of stroke [179].

There are two groups in which stroke symptoms can be classified: acute and long-term. Acute symptoms are especially crucial for revealing whether the patient will experience post-stroke disability [180]; therefore, a quick reaction to them is critical for maintaining a better quality of life [181]. Healthcare workers should pay attention to symptoms such as numbness, confusion, and dizziness, as well as general weakness, difficulty speaking, problems with coordination, and ophthalmological or even less common signs like vertigo, dysarthria, or partial sensory deficiency [182]. Acute stroke symptoms can persist beyond the initial event, potentially leading to long-term disabilities that may necessitate extended recovery periods or rehabilitation efforts. The most common symptoms include pain, anxiety, depression, and tiredness. It has been discovered that at least one-fourth of patients after a stroke will experience one or more of these symptoms [183,184,185].

Strokes are broadly classified into the more common ischemic type and the more lethal hemorrhagic type. The prevention of stroke in people with diabetes mainly focuses on lifestyle changes, such as quitting smoking, managing physical activity, and achieving weight loss [186], or through pharmacological and surgical interventions [187]. The combination of managing glucose levels, blood pressure (BP), and lipids along with the use of renin–angiotensin system (RAS) inhibitors, statins, and aspirin, has been shown to lower the risk of stroke [188].

#### 5.6.1. Ischemic Stroke

Clinical deterioration, the reason for ischemic stroke, arises from a possibly reversible inadequately perfused brain region known as the penumbra. If untreated, this region progressively evolves into irreversibly damaged tissue referred to as the core [189]. The type of ischemic stroke known as large vessel occlusion (LVO) stroke, responsible for over 50% of all stroke cases, occurs due to the blockage of major intracranial branches of the internal carotid artery, such as the proximal segments of the anterior and middle cerebral arteries or the vertebrobasilar arteries [190].

#### 5.6.2. Hemorrhagic Stroke

Hemorrhagic stroke results from bleeding within the brain due to the perforation of a blood vessel. It is classified based on the specific location of this vessel rupture into intracerebral hemorrhage (ICH) and subarachnoid hemorrhage (SAH) [191]. Diabetes and high blood glucose levels have a detrimental effect on people with these conditions, as they are connected with the risk of faster development of hematoma, frequent disability resulting from stroke, or death [192]. A post hoc analysis of the randomized Intensive Blood Pressure Reduction in Acute Cerebral Hemorrhage (INTERACT-2) experiment found that persistent hyperglycemia (>24 h) at the time of ICH occurrence was highly linked to low outcomes and significant disability (modified Rankin Scale [mRS] ≥ 3). In contrast, pre-existing diabetes was primarily a factor for residual disability [193]. Therefore, it should be considered that an intensive reduction in SBP in patients with suspected ICH may lead to a higher rate of hematoma expansion in individuals with hyperglycemia compared to those with normoglycemia [194].

### 5.7. Peripheral Artery Disease

Peripheral artery disease is one of the macrovascular complications of T2DM, increasing the risk of its occurrence. It has been proven that for every 1% increase in HbA1c, the chance of peripheral arterial disease (PAD) rises by as much as 30%, and it is most likely to display earlier in diabetic patients than in patients with euglycemia [195].

PAD is a chronic form of atherosclerosis that restricts blood flow to the lower limbs, leading to symptoms associated with reduced circulation. Initially asymptomatic, it may gradually lead to leg pain at rest. The pathognomonic sign of arterial insufficiency is claudication, which contributes to the deterioration of patients’ quality of life due to their progressively declining level of functioning [196]. Classic claudication can be defined as pain in the calf of one or both legs during exertion, such as walking. This pain does not occur at rest and typically subsides within a few minutes of standing or resting [197]. The prevalence of classic claudication among patients with symptomatic PAD over the past decade has been reported to range from 7.5% to 33% [198]. This variation appears to be influenced by factors such as age, with higher rates reported in older individuals with relevant clinical characteristics, including hypertension, a prior PAD diagnosis, or diabetes [199]. The progression of PAD, the frequency of its symptoms, and the elevated cardiovascular risks associated with systemic atherosclerosis serve as key measures of its impact. Among the various risk factors, T2DM plays a crucial role, second only to cigarette smoking, in its contribution to heightened susceptibility [200]. Approximately one-third of patients with claudication and half of those suffering from critical limb ischemia are affected by T2DM, underscoring its strong association with PAD severity [201]. The most painful manifestation of PAD is limb ischemia, characterized by pain in the lower limbs, impaired wound healing, and the development of skin ulcers [202], which can ultimately lead to amputation [203].

Patients with T2DM and PAD should be provided with comprehensive care focused on improving the peripheral blood flow and lowering the risk factors for cardiac events, including myocardial infarction, ischemic stroke, or cardiovascular death. The treatment includes structured programs for walking [204], smoking cessation, and weight management [205].

## 6. Conclusions

T2DM is a global health problem that affects more than 400 million people worldwide, and the number of people with diabetes continues to grow. The proper diagnosis of T2DM requires the exclusion of other types of diabetes, which is extremely important in determining prognosis and choosing a treatment method. T2DM is an inflammatory disease with a multifactorial etiology. The main pathomechanisms in which T2DM is developed are the defect of insulin production and IR in body tissues. Defective insulin synthesis may be attributed to the death of β-cells or β-cells dysfunction, which may be a result of several different mechanisms such as β-cells dedifferentiation, transdifferentiation, the induction of disallowed genes, the impact of oxidative or ER stress, as well as mitochondrial dysfunction. IR is a decline in tissue’s response to insulin. The main organs that play a role in IR are skeletal muscle, adipose tissue, and liver. Gut microbiota is another factor that may contribute to the development of diabetes; however, more studies are needed to conclude the importance of its role in this process. Recently, numerous effective medications have been introduced for the treatment of T2DM. These new therapies not only provide robust glycemic control but also avoid weight gain and the risk of hypoglycemia. As a result, they contribute to an improved quality of life for individuals with T2DM and its associated complications. It is also worth mentioning non-pharmacological methods of treatment, including appropriate physical activity, a balanced diet, and behavioral and psychological interventions. Possible complications of diabetes include chronic kidney disease, heart attack, stroke, and the development of neuropathy, retinopathy, as well as many others.

## Figures and Tables

**Table 1:** T2DM diagnostic criteria.

Hemoglobin A1C (HbA1C) ≥ 6.5%
or
Fasting plasma glucose ≥ 126 mg/dL
or
Plasma glucose after a 2-h 75-g oral glucose tolerance test (OGTT) of ≥200 md/dL
or
Random plasma glucose concentration of ≥200 mg/dL with classic symptoms of hyperglycemia or hyperglycemic crisis

**Table 2:** Results of the SURPASS-1 trial.

Outcomes		Tirzepatide 5 mg (n = 121)	Tirzepatide 10 mg (n = 121)	Tirzepatide 15 mg (n = 120)	Placebo (n = 113)
HbA1c (%)	baseline	7.97	7.88	7.88	8.08
	from baseline	−1.87	−1.89	−2.07	0.04
	versus placebo	−1.91	−1.93	−2.11	
Weight (Kg)	from baseline	−7.0	−7.8	−9.5	−0.7
	versus placebo	−6.3	−7.1	−8.8	

## Data Availability

The data used in this article were sourced from materials mentioned in References.
